# Cell cycle arrest is not yet senescence, which is not just cell cycle arrest: terminology for TOR-driven aging

**DOI:** 10.18632/aging.100443

**Published:** 2012-03-05

**Authors:** Mikhail V. Blagosklonny

**Affiliations:** Department of Cell Stress Biology, Roswell Park Cancer Institute, BLSC, L3-312, Buffalo, NY, 14263, USA

**Keywords:** senescence, geroconversion, gerosuppressants, rapamycin, mTOR

## Abstract

Cell cycle arrest is not yet senescence. When the cell cycle is arrested, an inappropriate growth-promotion converts an arrest into senescence (geroconversion). By inhibiting the growth-promoting mTOR pathway, rapamycin decelerates geroconversion of the arrested cells. And as a striking example, while causing arrest, p53 may decelerate or suppress geroconversion (in some conditions). Here I discuss the meaning of geroconversion and also the terms gerogenes, gerossuppressors, gerosuppressants, gerogenic pathways, gero-promoters, hyperfunction and feedback resistance, regenerative potential, hypertrophy and secondary atrophy, pro-gerogenic and gerogenic cells.

## INTRODUCTION

A year ago, I wrote a perspective “Cell cycle arrest is not senescence”, intended to clarify a new meaning of cellular senescence [1]. The perspective was not completely understood in part due to its title. The title was missing the word “yet”, which is now included. As discussed in the article, cell cycle arrest is not yet senescence and senescence is not just arrest: senescence can be driven by growth-promoting pathways such as mTOR, when actual growth is impossible. (This mechanism connects cellular senescence, organismal aging and age-related diseases, predicting anti-aging agents [2-6]). In brief, senescence can be caused by growth stimulation, when the cell cycle is arrested [7, 8]. As one hallmark, senescent cells loose proliferative potential (PP) - the potential to resume proliferation. Importantly, inhibitors of mTOR suppress hallmarks of senescence during cell cycle arrest so cells stay quiescent but not senescent [9-13]. Such quiescent cells, with inhibited mTOR, retain PP. Once again, cells may be arrested but retain PP, the ability to restart proliferation, when allowed. In certain conditions, p53 causes arrest but can preserve PP by inhibiting the mTOR pathway [14-16]. However, this phenomenon should not be misunderstood to indicate that “p53 induces proliferation or prevents arrest or keeps cells proliferating” or “arrested cells retain proliferation”; rather, p53 instead regulates proliferative potential. Although we tried to explain what p53 does exactly (causes arrest, while preserving PP) misunderstanding nonetheless ensued. One solution is not to use the term PP altogether, substituting for it the term RP (regenerative potential). In the organism, stem cells and wound-healing cells, while quiescent, are capable to regenerate tissues after cell loss. Unlike non-senescent cells, senescent cells cannot divide in response to cell loss and therefore lose the potential to regenerate tissues. In cell culture, quiescent cells preserve RP. If the cell cycle is blocked, activation of mTOR causes loss of RP [17]. New concepts need new terminology. Instead of squeezing novel meaning into the old terms, here we present new terms for a new meaning of the aging process. And a central term is gerogenic conversion or geroconversion.

### Geroconversion

**Figure 1:**
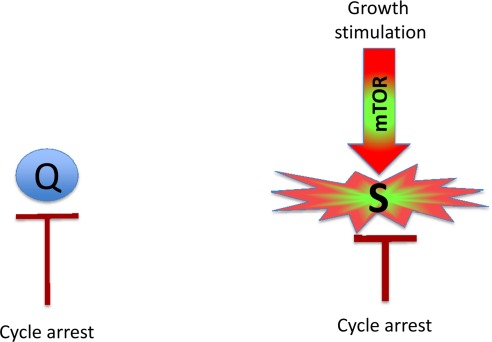
Quiescence versus senescence Q: quiescent cell. In the absence of the growth factors, normal cells undergo cell cycle arrest. S: senescent cell. When cell cycle is arrested, the growth signal (if it cannot reactivate cycling) drives senescence.

Mitogens and growth factors activate growth-promoting pathways, which stimulate (a) growth in size and (b) cell cycle progression. When cells proliferate, an increase in cell mass is balanced by division. Withdrawal of growth factors causes quiescence: the quiescent cell neither grows, nor cycles, and its functions and metabolism are low. In contrast, cell cycle blockage, in the presence of growth-stimulation leads to senescence (Figure 1). Hallmarks of senescence include a large flat morphology, senescence-associated beta-galactosidase (SA-beta-gal) staining, cellular over-activation and hyper-function, feedback signal resistance and loss of RP (that is, the inability to restart proliferation when the cell cycle inhibitor is removed). For example, in one well-studied cellular model, inducible ectopic expression of p21 causes cycle arrest (day 1) and senescence (after 3 days) [18, 19]. At first, the arrested cells are quiescent-like: they are not hypertrophic, and they are SA-beta-gal negative and retain RP. Thus, they can restart proliferation when p21 expression is switched off. After 3 days, however, the cells acquire a senescent morphology and, if p21 is then switched off, the cells cannot restart proliferation or die in mitosis [19]. Importantly, while inhibiting the cell cycle, p21 does not inhibit the mTOR pathway [8-17, 20, 21]. mTOR and perhaps some other growth-promoting pathways convert quiescence (day 1) into senescence (day 3). Inhibition of mTOR by rapamycin decelerates this ‘geroconversion’ [8-17, 20, 21].

Similarly other inhibitors of mTOR also suppress geroconversion [10, 11]. For example, in some cell lines, induction of p53 inhibits mTOR [22-26] and other anabolic pathways [27-32], thus suppressing geroconversion in cells arrested by ectopic p21 [14]. By itself, p53 causes cell cycle arrest but can suppress geroconversion [14-17, 33-35]. In cell culture, cell cycle arrest and geroconversion are initiated simultaneously. In proliferating cultured cells (especially cancer cells) mTOR is activated. Many agents cause cell cycle arrest without inhibiting mTOR (or other growth factor-sensing pathways). Once arrested, such cells are rapidly converted to senescent cells. This is accelerated geroconversion. So it may seem that senescence is “caused” by cell cycle arrest. The above examples, however, suggest that senescence is caused by growth-stimulation when the cell cycle is arrested.

In the organism, most cells are arrested and geroconversion can be slow. When chronically stimulated (but still arrested) they can become senescent. This physiological geroconversion can be imitated in cell culture [17].

The terms gerogenic conversion and oncogenic transformation sound alike.This is not a coincidence for choosing the term geroconversion. *Gerogenic conversion and oncogenic transformation are two sides of the same process*.

### Gerogenic oncogenes and gerogenes

**Figure 2:**
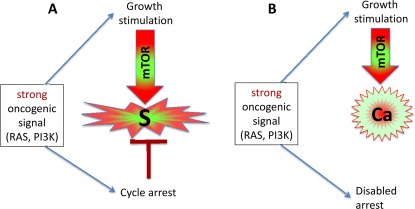
Strong oncogenic signaling, senescence and cancer (**A**) Strong mitogenic/oncogenic signal can simultaneously cause arrest and activate mTOR. Cells senesce. (**B**) Disabling of cell cycle control (loss of p16, Rb, p53) can convert senescence to cancer.

Activation of growth factor receptors, Ras and Raf family members and members of the MAPK and PI3K/Akt pathways are universal in cancer [36-38]. All these oncogenes activate the mTOR pathway [39-47]. They are gerogenic oncogenes, which drive the geroconversion of arrested cells. Because strong growth-promoting (mitogenic) signals induce cell cycle arrest [48-54], strong mitogenic signaling causes both conditions of senescence: arrest and mTOR/growth signal (Figure 2A). To avoid senescence, cancer cells must disable cell cycle control (Figure 2B) by either loss of p16, p53 and Rb or activation of c-myc, for example [36-38, 48, 55, 56]. In proliferating cells, gerogenic oncogenes render cells malignant and pro-gerogenic (see below). The same gerogenic oncogenes or their analogs accelerate aging and shorten life span in diverse species from worm to mammals. Therefore, these genes can be termed gerogenes [57]. Thus, the mTOR pathway shortens life span, whereas rapamycin extends life span [58-75]. Not coincidentally, Mutations that increase the life span of *C. elegans* inhibit tumor growth [76]. Finally, metabolic self-destruction, known as chronological senescence in yeast [60, 61, 77] is also stimulated by gerogenes and is inhibited by rapamycin [78].

### Gerosuppressors

Gerosuppressors are genes (and their products) that suppress geroconversion. Gerosuppressors (for example, PTEN, AMPK, sirtuins, TSC2, NF-1 and p53) antagonize the mTOR pathway (see for ref. [57]). Their inactivation shortens life span in model organisms. Gerosuppressors are also tumor suppressors. So gero-suppressors suppress both geroconversion and cancer.

### Gerosuppressants

Gerosuppressants are small molecules (such as rapamycin) that suppress geroconversion. Not co-incidentally, rapamycin also extends life span in diverse species from yeast to mammals. They can, in theory, be used to treat age-related diseases by slowing down aging, thus extending both maximal and healthy lifespan.

### Gero promoters

Small molecules or drugs that can accelerate or promote geroconversion. One potential candidate is phorbol esters, which can activate mTOR in some cells. Not surprisingly, it is also a tumor-promoter.

### Gerogenic pathways

Gerogenic signaling pathways promote geroconversion. Whether gerogenic pathways cause or abrogate cell cycle arrest is irrelevant. For example, strong mitogenic/growth signals can induce cell cycle arrest, instead of proliferation [48-54]. Simultaneously, in arrested cells, growth signals cause geroconversion, leading to senescence (Figure 2A). As another example, the effects of p53 on cell cycle and geroconverion can be dissociated [14].

### Pro-gerogenic conversion

In proliferating cells, overactivation of the mTOR pathway renders them pro-gerogenic. Cancer cells are proliferating pro-gerogenic cells. When such cells are forcefully arrested, they become senescent. Also, stimulation of mTOR in normal stem cells causes hyper-proliferation, pro-gerogenic conversion and cell exhaustion [79-84], contributing to aging.

### Gerogenic cell

Although loss of RP is very useful marker of senescence in cell culture, this marker may not play a key role in age-related pathologies in the organism, because most post-mitotic cells should not be able to restart proliferation anyway. (Notable exceptions are stem, wound-healing and satellite cells). I suggest that active mTOR_in arrested cells is a crucial marker of gerogenic cells and early senescence. Gerogenic (senescent) and quiescent cells can be distinguished by the levels of phosphorylated S6 (pS6), the ribosomal protein that is phosphorylated in response to mTOR activation: high in senescent cells and low in quiescent cells. Levels of pS6 in senescent cells may remain similar to the levels of pS6 in proliferating cells. So senescent/gerogenic cells have many features of proliferating cells. Interestingly, basal (fasting) levels pS6 were elevated in old mice [85]. Gerogenic cells could be defined as arrested cell with activated mTOR. The most physiologically relevant features are hypertrophy, hyperfunction and feedback resistance.

### Hypertrophy

Growth signals during cell cycle arrest lead to an enlarged cell morphology. From theoretical perspective, hypertrophy will eventually be limited by activation of lysosomes/autophagy [7]. This phenomenon may explain the activity of SA-beta-Gal, which is lysosomal enzyme [86-88] and active autophagy despite active mTOR [89, 90].

### Hyperfunction

Due to over-stimulation, senescent cells are hyperfunctional. For example, For example, senescent fibroblasts secrete many cytokines, growth factors and proteases (the hypersecretory senescence-associated secretory phenotype or SASP), senescent osteoclasts resorb bones, smooth muscle cells contract, platelets aggregate, neutrophils generate ROS, neurons charge, endocrine cells produce hormones. Noteworthy, SASP as a marker of senescence [91-99] is an example of hyperfunction.

### Feedback resistance

Overactivation of signaling pathways causes signal resistance due to feedback inactivation of the signaling pathway. As an example, mTOR/S6K overactivation causes insulin and GF resistance [100-104].

### Secondary atrophy

Hyperfunctions are associated with hypertrophy and hyperplasia. Yet, at the end, cells may fail either to function or to survive, leading to secondary atrophy. When cells fail, conditions become TOR-independent and terminal. This conceals hyperfunction as an initial cause, misleadingly presenting aging as a decline.

### From gerogenic cells to organismal aging

You might notice that an accumulation of molecular damage was never mentioned in this article. It was unneeded. Cellular aging and geroconversion is not caused by accumulation of random molecular damage. Although damage accumulates, I suggest that the organism does not live long enough to suffer from this accumulation with one special exception that illuminates the rule [105]. (The weakness of free radical damage theory was discussed in detail [106-114]).

One definition of organismal aging is an increase in the probability of death. Gerogenic cells (due to their hyper-activity and signal-resistance) may slowly cause atherosclerosis, hypertension, insulin-resistance, obesity, cancer, neurodegeneration, age-related macular degeneration, prostate enlargement, menopause, hair loss, osteoporosis, osteoarthritis, benign tumors and skin alterations. These conditions lead to damage -- not molecular damage but organ and system damage. Examples include beta-cell failure [115], ovarian failure (menopause) [116], myocardial infarction, stroke, renal failure, broken hips, cancer metastases and so on [6, 117]. These are acute catastrophes, which cause death. I suggest that by suppressing geroconversion, gerosuppressants will prevent diseases and extend healthy life span.
